# Hypoxia inducible factor-dependent upregulation of Agrp in glomus type I cells of the carotid body

**DOI:** 10.1016/j.molmet.2025.102095

**Published:** 2025-01-08

**Authors:** Luis Leon-Mercado, Ivan Menendez-Montes, Jonathan Tao, Bandy Chen, David P. Olson, C. Mackaaij, C.G.J. Cleypool, Laurent Gautron

**Affiliations:** 1 Center for Hypothalamic Research and Department of Internal medicine, UT Southwestern Medical Center, Dallas, TX, USA; 2 Division of Cardiology, Department of Internal Medicine, UT Southwestern Medical Center, Dallas, TX, USA; 3 Department of Pediatrics, University of Michigan Medical School, Ann Arbor, MI, USA; 4 Division of Surgical Specialties, Department of Anatomy, University Medical Center, Utrecht, the Netherlands

**Keywords:** Neuropeptide, Transcription, Cardiovascular adaptation, Hypoxia, Melanocortins

## Abstract

Agouti-related peptide (AgRP) is a well-established potent orexigenic peptide primarily expressed in hypothalamic neurons. Nevertheless, the expression and functional significance of extrahypothalamic AgRP remain poorly understood. In this study, utilizing histological and molecular biology techniques, we have identified a significant expression of *Agrp* mRNA and AgRP peptide production in glomus type I cells within the mouse carotid body (CB). Furthermore, we have uncovered evidence supporting the expression of the AgRP receptor melanocortin receptor 3 (Mc3r) in adjacent sympathetic neurons, suggesting a potential local paracrine role for AgRP within the CB. Importantly, AgRP immunoreactivity was also identified in glomus type I cells of the human CB. Given the unexpected abundance of AgRP in glomus type I cells, a chemoreceptor cell specialized in oxygen sensing, we proceeded to investigate whether *Agrp* expression in the CB is regulated by hypoxemia and associated oxygen-sensing molecular mechanisms. *In vitro* luciferase assays reveal that hypoxia stimulates the human and mouse Agrp promoters in a Hypoxia Inducible Factor (HIF1/2)-dependent manner. Our *in vivo* experiments further demonstrate that exposure to environmental hypoxia (10%) robustly induces *Agrp* expression in type I glomus cells of mice. Furthermore, these findings collectively highlight the hitherto unknown source of AgRP in murine and human type I glomus cells and underscore the direct control of *Agrp* transcription by HIF signaling.

## Highlights

• Agrp mRNA and AgRP peptides are produced by glomus cells of the carotid body.
• AgRP immunoreactivity is found in the human carotid body.
• In vitro, hypoxia stimulates the transcription of Agrp expression.
• Environmental hypoxia stimulates the transcription of Agrp expression in the carotid body.

## Graphical abstract

**Figure:**
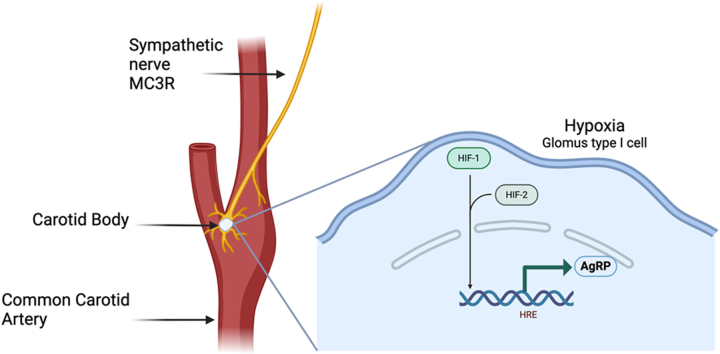

## 1 Introduction

Agouti-related peptide (AgRP) is a natural inverse agonist of melanocortin receptors 3 and 4 (Mc3r and Mc4r), counteracting the action of peptides derived from the Proopiomelanocortin gene (POMC) [[1], [2], [3]]. Within the central nervous system (CNS), AgRP is exclusively concentrated in a specific subset of neurons situated in the hypothalamic arcuate nucleus (Arc) [[3], [4], [5]]. Notably, Agrp mRNA expression in the Arc is markedly upregulated during periods of food deprivation and negative energy balance [[6], [7], [8], [9]], underscoring its pivotal role in regulating feeding behavior and metabolism [[10], [11], [12], [13], [14], [15]].

While research on AgRP within the CNS is extensive, there is limited evidence suggesting the presence of Agrp mRNA expression in the peripheral nervous system (PNS) and related structures. Early studies identified AgRP in the dorsal root ganglion (DRG) of rats [16,17], and transcriptomics studies have documented *Agrp* expression in spinal and vagal afferents [[18], [19], [20]]. Additionally, a recent study reported Agrp-driven Cre recombinase activity in colonic neurons of mice [21]. Nevertheless, AgRP production in these PNS sites remains uncertain. Beyond the nervous system, *Agrp* mRNA and its peptide have been observed in the adrenal medulla of both laboratory rodents and human subjects [1,[22], [23], [24]]. Furthermore, *Agrp* transcripts have been detected in the testis and lung [25,26], although confirmation of AgRP peptide production in these locations is lacking.

The primary objective of this study was to comprehensively map the distribution of AgRP and identify potential cell types responsible for its production in the PNS, with a focus on the carotid body (CB), a specialized cell type primarily responsible for oxygen sensing [27,28]. Consequently, our secondary aim was to investigate whether *Agrp* expression within the CB is directly regulated by hypoxia and oxygen-sensing molecular mechanisms. Our *in vivo* experiments demonstrate the upregulation of AgRP in the CB following exposure to hypoxic conditions. Additionally, our *in vitro* studies provide evidence that hypoxia stimulates *Agrp* expression through Hypoxia Inducible Factors (HIF1/2). This research unveils a previously undisclosed role for AgRP in the CB and underscores its potential involvement in oxygen sensing, enriching our understanding of its physiological significance in peripheral tissues.

## 2 Results

### 2.1 AgRP production in the mouse carotid body type I glomus cells

**Figure 1:**
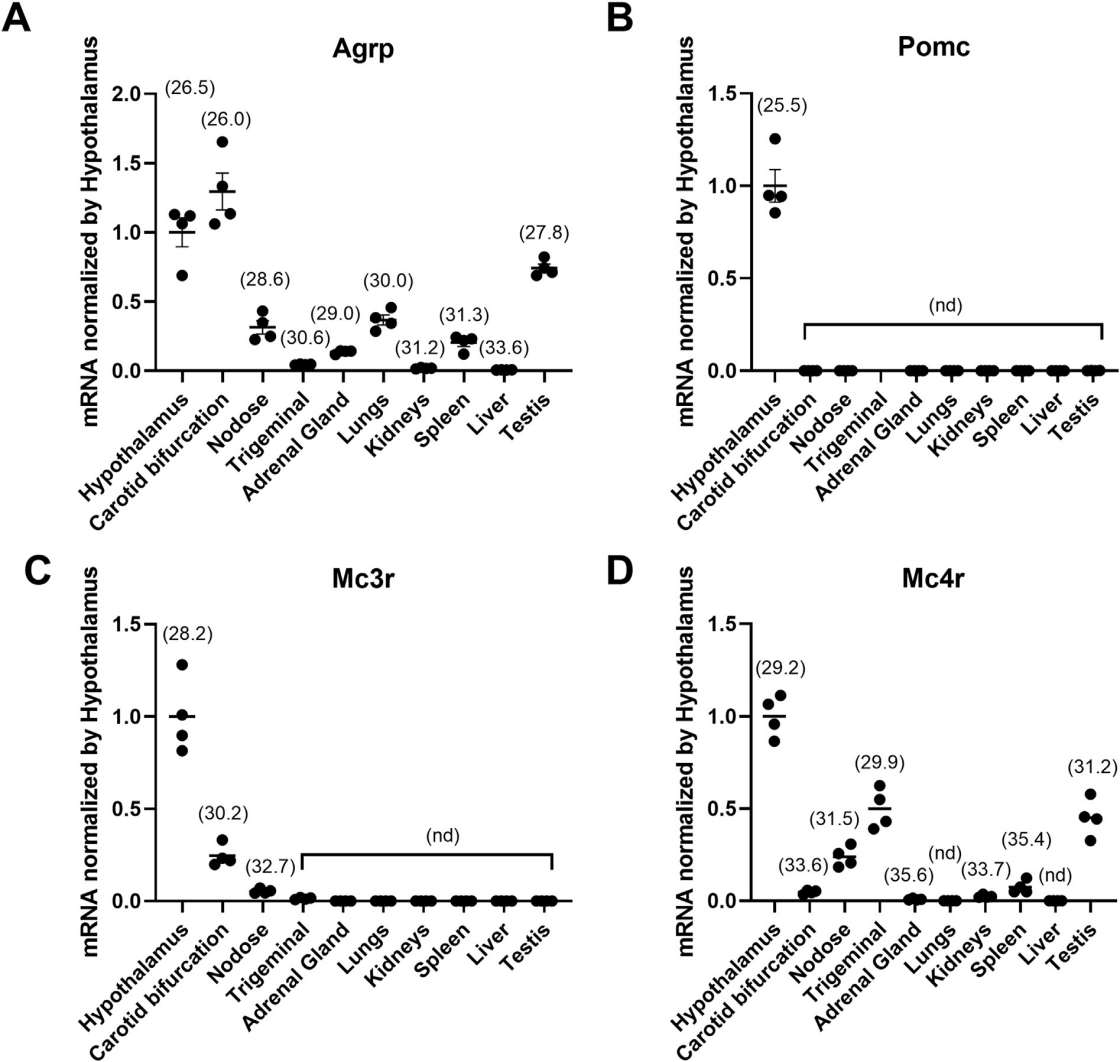
**Characterization of gene expression across peripheral tissues.** Relative mRNA expression of *Agrp* (**A**), *Pomc* (**B**), *Mc3r* (**C**), *Mc4r* (**D**), in hypothalamus, carotid bifurcation, nodose and trigeminal ganglia, adrenal gland, lungs, kidney, spleen, liver, and testis using qPCR in the C57BL/6J male mouse. Gene expression was normalized relative to the expression in the hypothalamus, and data are presented as mean ± S.E.M. (n = 4). Average Ct values are indicated above each bar graph. Abbreviation: nd, non-detectable.

Using quantitative polymerase chain reaction (qPCR) in C57BL/6J male mice, we initiated our investigation by examining potential *Agrp*-producing cells within both the hypothalamus and peripheral tissues including the carotid bifurcation, nodose and trigeminal ganglia, adrenal gland, lungs, kidney, spleen, liver, and testis. As expected, *Agrp* mRNA expression was prominently detected in the hypothalamus (Fig. 1A). More surprisingly, *Agrp* exhibited comparable expression levels in the carotid bifurcation to those in the hypothalamus (Fig. 1A). Moderate levels of *Agrp* expression were observed in the nodose ganglion, adrenal gland, lungs, spleen, and testis, while Agrp expression in the trigeminal ganglia, kidney, and liver remained markedly lower compared to the hypothalamus (Fig. 1A). Given AgRP's role in competing with POMC-derived peptides for binding to MC3R and MC4R within the CNS [[29], [30], [31], [32], [33]], we also investigated the expression of their corresponding genes. *Pomc* expression was exclusive to the hypothalamus, with no detection in other tissues (Fig. 1B). *Mc3r* displayed expression in both the hypothalamus and, to a lesser extent, the carotid bifurcation (Fig. 1C). The nodose ganglion exhibited very low levels of *Mc3r* expression, while it remained undetectable in other tissues. In contrast, *Mc4r* was primarily expressed in the hypothalamus, with moderate levels observed in sensory ganglia, but notably low or undetectable levels in the carotid bifurcation and other tissues (Fig. 1D).

**Figure 2:**
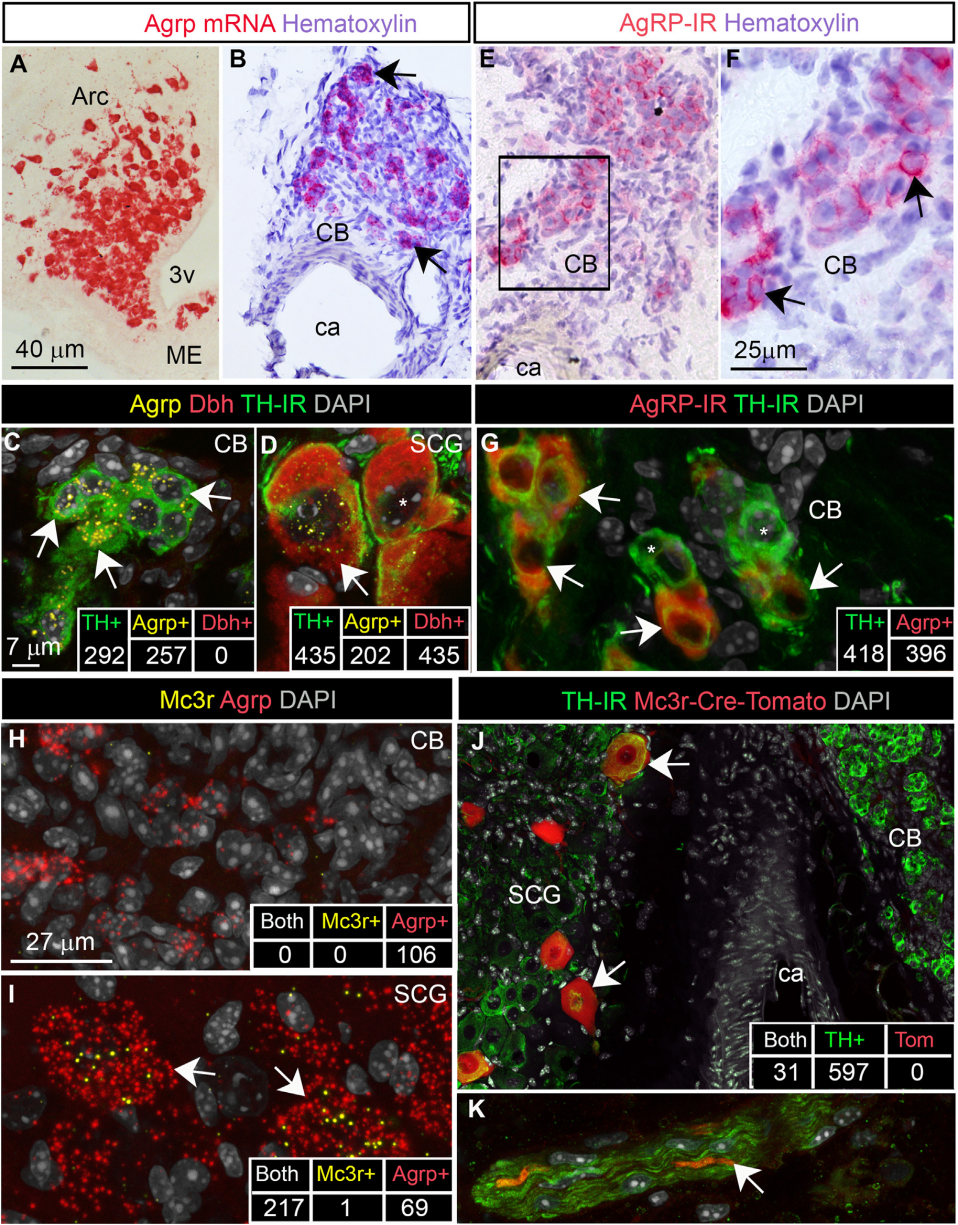
**Characterization of *Agrp*-expressing cells of the carotid bifurcation.** Chromogenic RNAScope in situ hybridization (ISH) for *Agrp* (red signals) in the Arc (**A**) and CB (**B**). Fluorescent ISH for *Agrp* (yellow) and *Dbh* (red) in combination with TH immunoreactivity (green) in the CB (**C**) and SCG (**D**). White arrows indicate cells expressing *Agrp* and TH. Numbers of labeled cells are indicated in the insets. Chromogenic AgRP immunoreactivity (red) in the CB (**E, F**). Double staining for AgRP and TH in the CB (**G**). White arrows indicate cells positive for AgRP and TH. Numbers of labeled cells are indicated in the insets. Fluorescent ISH for *Agrp* (red) and *Mc3r* (yellow) in the CB (**H**) and SCG (**I**). White arrows indicate cells expressing *Agrp* and *Mc3r*. Numbers of labeled cells are indicated in the insets. TH immunostaining (green) of the carotid bifurcation from Mc3r-Cre-Tomato mice (red) (**J, K**). White arrows indicate cells positive for TH and Tomato. Numbers of labeled cells are indicated in the insets. Nerve containing TH and Tomato-positive fibers (**K**). Chromogenic and fluorescent tissues were counterstained with hematoxylin (purple) and DAPI (grey), respectively. Abbreviations: 3v, third ventricle; Arc, arcuate; c, carotid artery; CB, carotid body; ME, median eminence; IR, immunoreactivity. Scale bar in A applies to B and C. (For interpretation of the references to color in this figure legend, the reader is referred to the Web version of this article.)

Given the complex nature of the carotid bifurcation, comprising components like the sympathetic superior cervical ganglion (SCG), CB, and various autonomic nerves [34], we employed molecular histology techniques to identify the specific cell types expressing *Agrp*. Using RNAScope in situ hybridization (ISH), we confirmed strong ISH signals in Arc neurons proximal to the third ventricle (Fig. 2A). Concurrent with our qPCR findings, abundant *Agrp* mRNA signals were evident in the CB (Fig. 2B). Remarkably, *Agrp*-expressing cells, resembling those of type I glomus cells displayed clustered labeling patterns (Fig. 2B). We utilized immunohistochemistry (IHC) to identify tyrosine hydroxylase (TH), the rate-limiting enzyme in dopamine synthesis, as a marker for type I glomus cells within the CB [35,36]. Conversely, the SCG is characterized by low dopamine levels and an abundance of noradrenergic cells, identifiable through the presence of dopamine beta-hydroxylase (*Dbh*). Extending our analysis with Multiplex fluorescent ISH in combination with IHC, we established the presence of *Agrp* transcripts in approximately 88% of type I glomus cells (Fig. 2C). These cells were distinguished by their TH-positivity and *Dbh*-negativity. Additionally, *Agrp* transcripts were detected in approximately 50% of neurons within the SCG adjacent to the CB (Fig. 2D). In comparison to type I glomus cells, SCG neurons were larger and *Dbh*-positive. Consistent with our ISH findings, AgRP immunoreactivity followed a distribution pattern in the CB that closely mirrored that of *Agrp* mRNA (Fig. 2E,F). Double IHC analysis confirmed that approximately 95% of glomus cells were also AgRP-immunoreactive (Fig. 2G). However, the SCG exhibited only very faint AgRP immunoreactivity, primarily localized within the soma of sympathetic neurons (Supplementary Fig. 1). Thus, the extent of AgRP peptide production by SCG neurons remained uncertain. In fact, Agrp mRNA was often detectable in peripheral sites not exhibiting any immunoreactivity for AgRP peptide (Supplementary Fig. 1).

Using RNAScope, *Mc3r* signals were notably absent in type I glomus cells (Fig. 2H) but were observed in approximately 75% of *Agrp*-positive SCG neurons (Fig. 2I). *Mc3r*-expressing cells constituted approximately 12% of all TH-positive SCG neurons. To validate these findings, we utilized the Mc3r-Cre-tdTomato reporter mouse model [37], which confirmed the presence of tdTomato in a subset of SCG neurons but not in type I glomus cells (Fig. 2J). Intriguingly, presumptive sympathetic axons co-expressing TH and tdTomato were occasionally observed traversing the carotid bifurcation (Fig. 2K). These observations suggest that AgRP derived from the CB may exert paracrine effects on nearby sympathetic neurons, dependent on MC3R signaling.

### 2.2 Hypoxia-induced stimulation of *Agrp* transcription in an HIF-dependent mechanism

**Figure 3:**
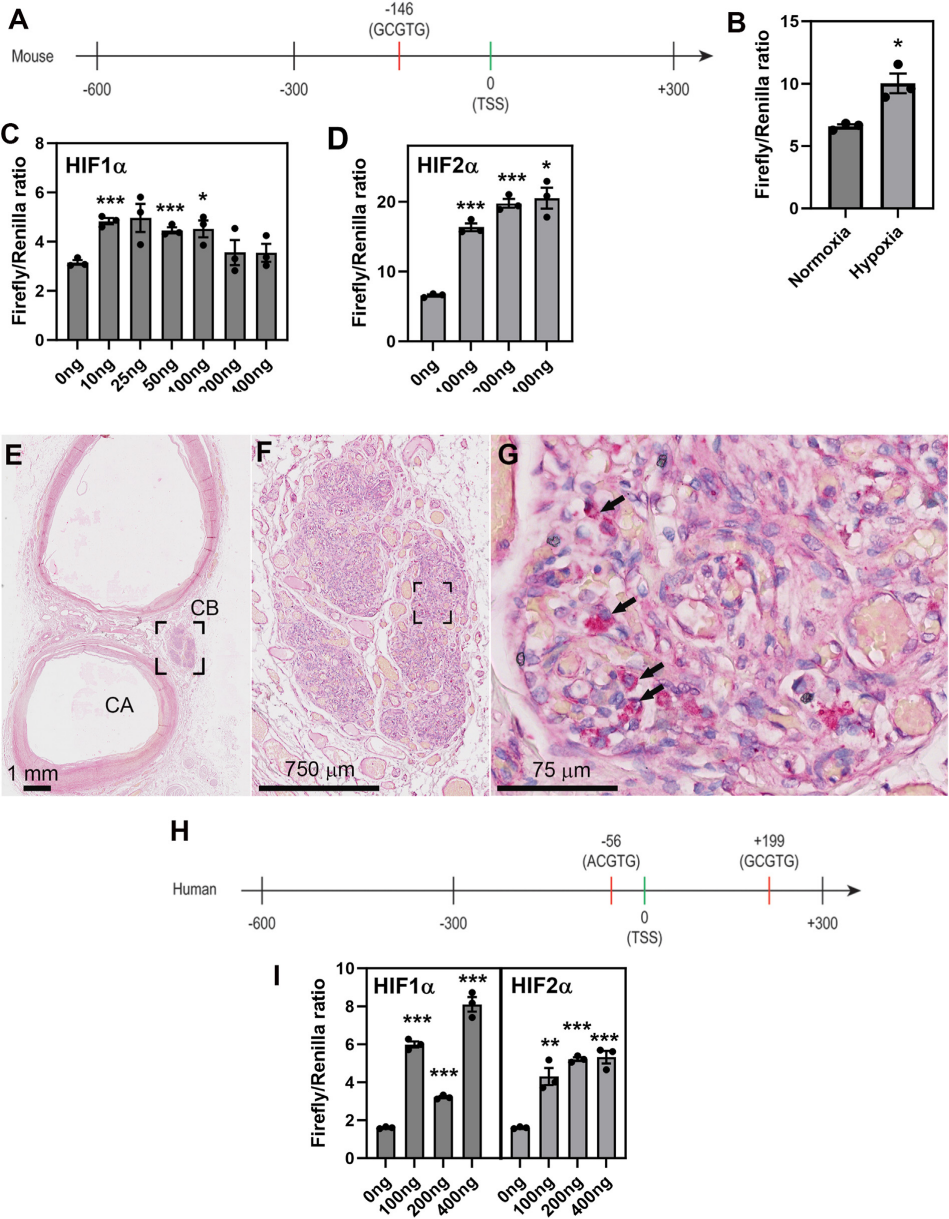
**Effects of hypoxia and HIF signaling on *Agrp* transcription in HEK293 cells and human data.** (**A**) HREs in the mouse *Agrp* promoter. (**B**) Exposure to hypoxia results in 1.5-fold increase in luciferase activity compared to normoxia transfected with the Agrp mouse promoter. (**C**) HIF1α- and HIF2α-dependent (**D**) luciferase activity in cells transfected with different amounts of plasmid and the Agrp mouse promoter. Data are expressed as absolute results (left) or normalized to the 0 ng group (right). (**E, F, G**) Representative microscopic image of a human CB (PPFE slice from case # 1 left side) stained with an antibody against AgRP. Immunoreactivity (chromogenic FastRed) is seen in a subset of CB cells resembling type I glomus cells (black arrows). Tissue was counterstained with hematoxylin. (**H**) HREs in the human *Agrp* promoter. (**I**) HIF1α- and HIF2α-dependent luciferase activity in cells transfected with different amounts of plasmid and the Agrp mouse promoter. Data are expressed as absolute results (left) or normalized to the 0 ng group (right). Data are the mean ± S.E.M. T-test results are as ∗, p < 0.05; ∗∗, p < 0.01; ∗∗∗, p < 0.001. Abbreviations: CA, carotid artery; CB, carotid body.

The role of hypoxia in directly stimulating *Agrp* transcription through a mechanism dependent on Hypoxia-Inducible Factors (HIFs) was investigated. HIF1α and HIF2α, known as Hypoxia-inducible factors-1 and -2, play a pivotal role in enabling various cell types, including type I glomus cells, to sense and respond to hypoxemia, thus serving as master regulators of oxygen homeostasis in the body [38]. Building upon our anatomical observations, we formulated the hypothesis that *Agrp* transcription is directly regulated by hypoxia through an HIF-dependent pathway. Supporting this hypothesis, Hypoxia-Responsive Elements (HREs) with consensus sequence GCGTG were found within the mouse *Agrp* promoter (Fig. 3A). Furthermore, luciferase assays conducted *in vitro* revealed that exposure to hypoxia (5% O_2_ for 24 h) led to a 1.5-fold increase in *Agrp* transcription in HEK293 cells that were transfected with the mouse *Agrp* promoter (Fig. 3B). When cells were transfected with HIF1α (Fig. 3C) or HIF2 (Fig. 3D), transcription from the mouse *Agrp* promoter was significantly elevated, up to 1.5-fold and 3-fold, respectively.

Whether the previous observations apply to humans was tested subsequently. Using an antibody against AgRP previously validated in human tissues by the Human Protein Atlas [39], we observed AgRP immunoreactivity in human CBs (Fig. 3E–G; Supplementary Fig. 2). AgRP immunoreactivity was noticed in cells resembling type I glomus cells based on their distribution and shape. Additional histological images can be found in Supplementary Fig. 2. Human HREs were also found within the human *Agrp* promoter (Fig. 3H). Additional luciferase experiments demonstrated an increase in the human *Agrp* transcription by up to 5-fold or 3-fold in cells transfected with either HIF1α or HIF2α, respectively (Fig. 3I). Collectively, these findings substantiate that *Agrp* is a direct target gene of HIFs in both mice and humans.

### 2.3 Environmental hypoxia-induced regulation of Agrp transcription is tissue-dependent

**Figure 4:**
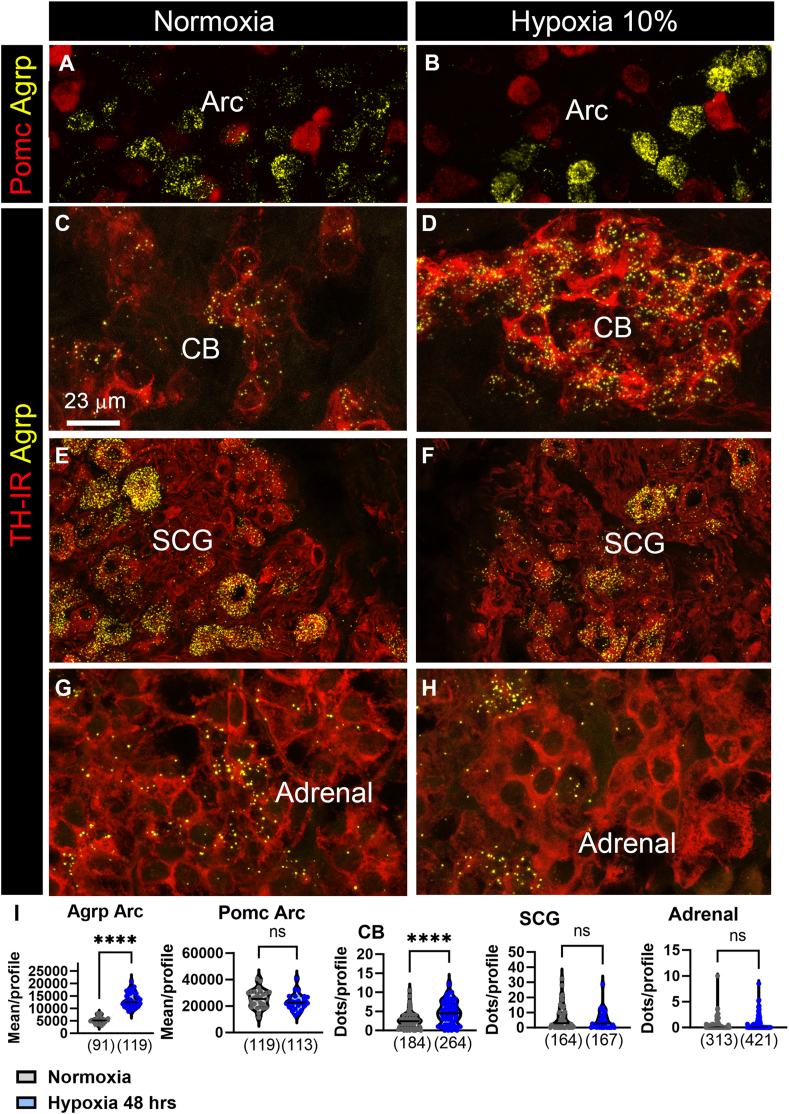
***Agrp* regulation in response to hypoxia.** RNAScope in situ hybridization (ISH) for *Agrp* (yellow) and *Pomc* (red) in the Arc of wild-type mice in response to normoxia (**A**) and 48 h hypoxia (**B**). *Agrp* (yellow) and TH (red) in the CB (**C, D**), SCG (**E, F**), and adrenal medulla (**G, H**) in response to normoxia and 48 h hypoxia. Estimates of Agrp expression in the above tissues in response to normoxia (grey dots) and 48 h hypoxia (blue dots) (**I**). Each dot represents one data point, and the number of counted cells is indicated below each bar graph. Data were analyzed using an unpaired T-test with P < 0.05 as statistically significant. Abbreviations: see Figure 2. (For interpretation of the references to color in this figure legend, the reader is referred to the Web version of this article.)

Type I glomus cells within the CB are specialized in sensing acute changes in systemic oxygen levels and play a crucial role in coordinating cardio-metabolic responses to hypoxia [27,28]. Prolonged exposure to hypoxemia leads to significant morphological and transcriptional adaptations within the CB, which are essential for maintaining systemic oxygen levels [27,28]. Given these observations and our previous *in vitro* data, we postulated that *Agrp* expression levels in the CB might be influenced by environmental levels of oxygen. To test this hypothesis, a cohort of mice was housed in a sealed chamber with a constant oxygen concentration of 10% for 48 h, and their *Agrp* expression profiles were compared to mice housed under normal ambient oxygen levels. Within the Arc, we observed a substantial increase in *Agrp* expression in hypoxic mice relative to the control group (Fig. 4A,B). In contrast, *Pomc* expression levels remained unchanged between the two groups (Fig. 4A,B). Similarly, in mice exposed to hypoxia, *Agrp* signals were notably higher in TH-positive glomus cells compared to the normoxic group (Fig. 4C,D). Remarkably, TH immunoreactivity itself was visibly enhanced in type I glomus cells of hypoxic mice (Fig. 4C,D), consistent with previous findings [35,36]. Interestingly, *Agrp* signals remained unaltered in TH-positive cells of both the SCG (Fig. 4E,F) and adrenal medulla (Fig. 4G,H) within the same group of mice. Quantitative analysis of *Agrp* expression by ISH confirmed a significant upregulation of *Agrp* signals in the Arc and CB of hypoxic mice but not in the SCG or adrenal glands (Fig. 4I). These results demonstrate that hypoxia exerts a regulatory influence on *Agrp* transcription, albeit in a tissue-specific manner.

In the hypothalamus, changes in hormonal signals, such as insulin, leptin, and inflammatory cytokines, are well known to stimulate *Agrp* expression during periods of food deprivation [[6], [7], [8], [9]]. However, circulating levels of glucose, insulin, leptin, and TNFα remained unchanged in hypoxic mice after 48 h (Supplementary Fig. 3). In contrast, blood lactate was significantly elevated in the hypoxia group (Supplementary Fig. 3). Additionally, food deprivation did not stimulate *Agrp* in type I glomus cells and, furthermore, type I glomus cells were devoid of the leptin receptor (Supplementary Fig. 4). Together, our data suggest that the elevated *Agrp* expression observed in the Arc and CB of hypoxic mice was unrelated to the hormonal and inflammatory changes typically associated with food deprivation. Instead, *Agrp* transcription is directly regulated by signals associated with hypoxia.

## 3 Discussion

AgRP action has been largely studied in hypothalamic neurons, however, the regulation and function of AgRP outside the CNS remains largely unexplored. Our evaluation of AgRP expression across different tissues showed an unexpected enrichment of *Agrp* mRNA and AgRP protein in type I glomus cells of the CB. Low mRNA expression was found in the nodose ganglion, adrenal gland, lungs spleen and testis compared to the hypothalamus. The CB was the only tissues with consistent *Agrp* mRNA and peptides and, consequently, we decided to focus our work on the CB in response to hypoxemia.

Our *in vivo* experiments show that hypoxia-induced upregulation of *Agrp* occurred in type I glomus cells and Arc neurons, but not in the SCG and adrenal medulla. This is consistent with our *in vitr*o data showing the *Agrp* promoter to be upregulated by hypoxia in a HIF-dependent manner, and the fact that few genes are transactivated by hypoxia across all cell types [40]. Even though Hif1 and 2 are widely expressed across the body, due to interactions with different cofactors, each cell type uniquely responds to hypoxia [40]. The transcription control of *Agrp* has been extensively studied in hypothalamic neurons in the past [9,41]. However, to our knowledge, there is no published data on the response of Agrp to hypoxia before this study [42]. That said, Hif-1 upregulation has been reported in the Agrp neurons of food deprived mice [9], thus suggesting that HIF signaling may play a role in fasting-induced *Agrp*. Prior studies indicated that HIF signaling stimulated *Pomc* transcription, but we couldn't confirm this observation in our mice [43,44].

Interestingly, exposure to decreased levels of environmental oxygen leads to anorexia of unexplained origin in humans [45,46] and rodents [47,48]. Our finding that *Agrp* is elevated following hypoxia is surprising given that *Agrp* expression in hypothalamic neurons is normally elevated in starving animals [[49], [50], [51]]. However, elevated hypothalamic *Agrp* has been reported in other models of anorexia in the past [52]. More importantly, AgRP peptide and AgRP neurons also exert a profound inhibitory effect on oxygen consumption [13,53,54] and hypoxia is associated with lowered oxygen consumption [55,56]. Thus, it is possible that hypoxia-induced *Agrp* may contribute to the latter adaptive phenotype.

At the level of the CB, it is also deduced that AgRP may serve as a paracrine signal between type I glomus cells and nearby MC3R-bearing sympathetic neurons. It is also remarkable that cells identified to produce *Agrp* mRNA in the periphery are all cells that express sympathoadrenal genes [57]. For instance, within the adrenals, AgRP plays a role in modulating catecholamines and corticosteroids secretion [22,23], however, the physiological role of AgRP in other peripheral organs is yet to be described. While it is difficult to predict the exact membrane effects of AgRP on SCG neurons, it has been reported that MC3R signaling causes neuronal firing [41,58,59]. Because AgRP is an inverse agonist of the MC3R [60], the CB-derived AgRP may inhibit the electrophysiological activity of sympathetic MC3R-expressing neurons, thereby changing the sympathetic outflow to structures involved in cardio-respiratory functions [61]. It may also be that AgRP is released in the bloodstream to act on distant targets and/or MC3/4Rexpressing sensory neurons involved in cardio-respiratory functions. Further functional studies are therefore warranted to test these hypotheses. Moreover, it would be interesting to test whether AgRP from the CB competes with circulating gamma-MSH, a high affinity MC3R agonist. This research sheds new light on the intricate regulatory network involving AgRP in oxygen sensing within the CB, expanding our understanding of its potential physiological significance.

Based solely on our findings, it is not possible to say what is the exact role of CB-derived AgRP. The central and peripheral AgRP effects are likely independent considering that AgRP doesn't cross the blood–brain barrier very effectively [62]. Nonetheless, it is likely that secreted AgRP may be a new molecular player in CB-related functions including modulation of cardiovascular and respiratory functions [[63], [64], [65]]. Since we found AgRP in the human CB, it is tempting to speculate that AgRP at the level of the CB may play a role in adaptations to chronic hypoxia resulting from sleep apnea, heart failure, respiratory infections, and other hypoxic conditions with subclinical symptoms in humans. For example, obese individuals frequently contend with episodes of systemic intermittent hypoxia [66], and obese males also exhibit elevated circulating AgRP [67,68]. If indeed plasma AgRP reflects episodes of hypoxia, a possibility that necessitates further validation, it would be interesting to assess whether elevated serum levels of AgRP could serve as a potential biomarker for chronic hypoxia. Recent evidence indicates that the CB can cause hypertension by prompting the sympathetic outflow [69]. Therefore, the interaction of AgRP and gamma-MSH in the CB may also have implications for the regulation of blood pressure. Gamma-MSH, for example, has been shown to significantly affect cardiovascular function, high sodium diets increase gamma-MSH and hypertension can be a specific consequence of impaired POMC processing into gamma-MSH [70]. Further studies are also warranted to better study the link between melanocortin signaling and altitude sickness. Similarly, it has been recently speculated that a failure of CB to adapt to hypoxia may exaggerate the severity of Covid19 [71]. Thus, more studies are warranted to investigate how AgRP signaling in the CB and connected neurons is pertinent to numerous hypoxic conditions.

## Declaration of generative AI and AI-assisted technologies in the writing process

None to declare.

## CRediT authorship contribution statement

**Luis Leon-Mercado:** Writing – review & editing, Writing – original draft, Visualization, Validation, Methodology, Investigation, Formal analysis, Data curation, Conceptualization. **Ivan Menendez-Montes:** Writing – review & editing, Validation, Resources, Investigation, Formal analysis, Data curation, Conceptualization. **Jonathan Tao:** Methodology, Investigation, Formal analysis. **Bandy Chen:** Investigation, Formal analysis. **David P. Olson:** Writing – review & editing, Resources. **C. Mackaaij:** Visualization, Investigation. **C.G.J. Cleypool:** Writing – review & editing, Visualization, Resources, Investigation. **Laurent Gautron:** Writing – review & editing, Writing – original draft, Visualization, Validation, Supervision, Resources, Project administration, Investigation, Funding acquisition, Formal analysis, Data curation, Conceptualization.

## Declaration of competing interest

The authors declare no competing interests.

## Data Availability

Data will be made available on request.

## STAR Methods

**Mice.** Mice included in this study were adult males (6- to 8-week-old) on a C57Bl/6J genetic background obtained from the Jackson Laboratory (#000664). Mc3r-Cre-Tomato mice from Michigan (David Olson) [72]. Agrp-Cre-Tomato mice were produced by crossing AgRP-ires-Cre mice (Jackson Laboratory stock #012899) with B6.Cg-Gt(Rosa)26Sortm14(CAG-td-Tomato)Hze/J (Jackson Laboratory stock #007914). Mice had ad libitum access to tap water and a commercial rodent chow (Harlan Teklad TD.2016 Global) unless indicated otherwise. They were housed in a light-controlled (12 h on/12 h off) and temperature-controlled environment (22 °C) in a barrier facility. The procedures were approved by the Institutional Animal Care and Use Committee of the University of Texas Southwestern Medical Center at Dallas (APN#2021-103086). Animal sacrifices were conducted during the light phase between 10 AM and 2 PM. Procedures were performed in accordance with the ARRIVE guidelines on animal experimentation. Moreover, per the Sex and Gender Equity in Research (SAGER) guidelines, we would like to report that only male mice were included in our study. Since no previous studies have examined sex differences in the mouse carotid body, it remains unclear whether sex is relevant to the interpretation of our results.

**Perfusion and mouse tissues preparation**. For histology, mice were anesthetized with an overdose of chloral hydrate (500 mg/kg, i.p.). Due to the small size of the mouse CB, anesthetized mice were administered Evans blue dye following the method of McDonald and Blewett (McDonald, 1981 #32008) to allow proper identification and collection of tissue. In brief, 400 μl of Evans blue dye prepared in sterile saline was injected in anesthetized animals by cardiac puncture. Immediately thereafter, 1 ml of 10% formalin (Sigma) was administered by the same route. We removed the carotid bifurcation and, with the help of a dissection microscope and fine forceps, a dark blue spot corresponding to the CB was resected. Brain and adrenals were also collected from the same mice. Samples were fixed at 4 °C in formalin for 24 h and immersed in 30% sucrose for another 24 h, prior to freezing. Using a cryostat, 14 μm thick tissue sections were collected on SuperFrost slides and stored at −80 °C until needed.

**Human tissues studies**. Single sided CBs of three human cadavers (3 females, age between 85 and 87 years) were examined microscopically in tissue sections of the carotid bifurcation, as previously described [73]. The subjects entered the Anatomy Department of the University Medical Center Utrecht, the Netherlands, through a body donation program. Informed consent was obtained prior to death. Sex assigned at birth of our subjects only included females, for lack of samples from males. It is not known whether sex is relevant to the interpretation of our results. Whole body perfusion was performed via the femoral artery with 3% formaldehyde solution. Bodies were stored at 4 °C for 4-6 months prior to their use. Carotid bifurcations with both the internal and external carotid arteries and surrounding connective tissue were resected and stored in 0.1M phosphate buffer (pH7.4) containing 15% sucrose and 0.05% sodium azide. Samples were then decalcified by immersion in 12% (w/v) EDTA disodium salt (Merck, Darmstadt, Germany) in distilled water at pH7.4 for 13 days and further processed for paraffin embedding by immersion in graded series of ethanol and xylene (for more details on the technical procedures see [73]).

Using a Histocore Autocut R (Leica Nussloch, Germany) microtome, 5 μm thick paraffin sections of the presumably carotid bifurcation area containing the CB were cut and collected on glass slides, air dried and fixed using a slide drying table (Medax, Kiel, Germany) at 60 °C for 2 h. Routine hematoxylin and eosin staining was performed to confirm CB tissue presence. Sections were then immunohistochemically stained for the presence of AgRP, by using antibodies raised against AgRP.

All sections were dewaxed in xylene, rehydrated to water through a series of graded ethanol followed by antigen retrieval in citrate buffer (pH6.0) or Tris/EDTA buffer (pH 9.0, DBH/AgRP) for 20 min at 95 °C. All washing steps were performed with 0.05M Tris Buffered Saline (pH 7.6) + 0.05% Tween20 (TBS-T) buffer. Sections were pretreated with 5% Normal Human Serum (NHS) for 10 min at Room Temperature (RT) before incubation with the primary antibody rabbit anti-AgRP (HPA041017, Sigma, Germany, 1:100 in TBS-T +5% BSA, overnight 4 °C). This primary antibody was validated in the Human Protein Atlas [39] and ranked as a reliable antibody with “high consistency between antibody staining and RNA expression data”. After washing in 3 changes of TBS-T to remove all unbound antibodies, sections were incubated with Brightvision Poly Goat-anti-Rabbit -Alkaline phosphatase (Immunologic, Duiven, The Netherlands) for 30 min at RT. Immunoreactivity was visualized with Liquid Permanent Red (LPR, Dako, Glostrup, Denmark) for 10 min at RT, followed by counterstaining in Hematoxylin. Slides were airdried for 15 min at 60 °C and cover slipped with Entellan (Merck, Darmstadt, Germany). Human adrenal gland sections were used as positive controls for AgRP. Negative controls were obtained by incubation of sections of the carotid bifurcation, adrenal gland, vagus nerve and sympathetic trunk with Tris-buffered saline with 3% bovine serum albumin without primary antibodies.

Of note, we performed RNAscope on the human CB to detect human Agrp transcripts, but failed to detect any signals, likely due to excessively long post-mortem intervals. Fresh carotid samples would be more amenable to mRNA-studies, but such samples are exceedingly difficult to obtain.

**QPCR assays.** C57Bl/6J mice were anesthetized with an overdose of chloral hydrate (500 mg/kg, i.p.) and decapitated. Tissues (hypothalamus, carotid bifurcation, nodose ganglion, trigeminal ganglion, adrenals, lungs, kidneys, liver, and testis) were rapidly collected in RNA PureZol (Bio-Rad, Inc). RNA concentration was assessed by UV spectroscopy using the Beer–Lambert law at 260 nm wavelength. Complementary DNA from 1 μg of input RNA was generated with the High-Capacity cDNA Reverse Transcription Kits (Life Technologies). cDNA was diluted in ultra-pure distilled water, DNase-free RNase free (Invitrogen, 10,977,015). Transcript levels were measured in duplicate using TaqMan Universal PCR Master Mix (Applied Biosystems, 4,304,437), the ABI PRISM 7900HT Sequence Detection System (Applied Biosystems, 4,317,596) and the CFX maestro software. Relative gene expression was calculated by the 2-ΔΔCT method. Taqman primers used in this study included Agrp (Mm00475829), Pomc (Mm00435874_m1), Mc3r (Mm00434876_s1), and Mc4r (Mm00457483_s1). Normalizer was Gapdh (Mm99999915_g1). Hypothalamic samples were normalized to 1 and data were expressed as average mean with s.e.m.

**Environmental hypoxia.** Mice were housed inside a sealed chamber (Coy Laboratory Products, Inc., Grass Lake, MI) with O_2_ levels at 10% for 48 h. Control normoxic mice were housed in a similar chamber with O_2_ levels at 21%. Hypoxia was chosen based on literature indicating that 10% O_2_ levels cause significant morphological and transcriptional plasticity of the mouse CB without ill effects [74].

**Luciferase assays.** Luciferase assays were performed in HEK293 cells (CRL-3216, ATCC) using Dual-Luciferase Reporter Assay system (E1910, Promega). Eighty % confluent p24 wells were transfected with the following amounts of plasmids per well: 5 ng of renilla luciferase reporter plasmid, 400 ng of pGL4 firefly luciferase reporter plasmid containing the corresponding *Agrp* promoter sequence and different amounts of HIF1α or HIF2α expression plasmids. In order to keep the total amount of plasmid equal under different conditions, a plasmid expressing neomycin resistance was added to reach a total amount of 805 ng of total plasmid per well. Cells were harvested 48h after transfection following manufacturers protocol. For hypoxia exposure experiments, no HIF plasmids were used. 24h post-transfection, cells were exposed to 5% oxygen for 24h before performing the luciferase assay.

**RNAScope and mouse immunohistochemistry**. RNAScope and immunohistochemistry were used to survey the distribution of *Agrp* mRNA and AgRP peptide, respectively. Chromogenic and fluorescent in situ hybridization (ISH) were performed using the RNAscope® 2.5 HD Red Assay (Advanced Cell Diagnostics; cat#322360) and RNAscope® Multiplex Fluorescent Detection Kit v2 (Advanced Cell Diagnostics, cat#323110), respectively. Briefly, slides were baked at 60 °C for 30 min, pretreated following the manufacturer's instructions, and probes were applied for 2hrs at 40 °C. Probes were Agrp (#400711-C2), Pomc (#314081-C3), Lepr (#402731), and Dbh (#407851-C1). Amplification steps were performed exactly as directed by the manufacturer. In the case of chromogenic ISH, FastRed accumulated to form red dots and slides were covered with EcoMount (Biocare medical). In the case of fluorescent ISH, detection relied on Opal dyes (Opal 520 #1487001 KT and Opal 570 #FP1 1488001 KT, Akoya biosciences). Fluorescent samples were counterstained with DAPI and covered with ProLong Gold Antifade (ThermoFisher, cat#P36930). Following RNAScope, immunohistochemistry (IHC) for tyrosine hydroxylase (TH) was done by incubating slides with a chicken anti-TH antibody (1:1000) (Aves lab; TYH) and AlexaFluor 488 anti-chicken (1:500) (Invitrogen, cat# A11039 AB152). The characterization of this antibody can be found elsewhere [75]. IHC for AgRP was done by incubating slides with rabbit anti-AgRP antibody (1:2000) (Abcam, cat# ab113481) overnight, followed by 2h incubation with Horse Anti-Rabbit IgG Polymer Detection Kit, Alkaline Phosphatase (Vector labs, Cat# MP-5401), for the chromogenic reaction, the ImmPACT Vector Red Substrate Kit, Alkaline Phosphatase (AP) was used as recommended by the vendor (Vector labs, cat# SK-5105). According to the manufacturer, the AgRP antibody recognizes a single band at 14 kDa on human brain lysates processed for western blot. In our hands, this antibody labeled AgRP-positive fibers in the mouse brain following a pattern entirely consistent with the published literature using other antibodies.

**Imaging.** Bright-field images of mouse tissues were captured using a Zeiss Axioplan light microscope attached to a digital camera. Brightfield images (40x magnification) and tilescans (10× magnification) of human tissues were captured using a DM6 microscope, DFC7000 T camera and LASX software (all Leica). Immunofluorescence images were captured using a Zeiss LSM880 confocal microscope available at UT Southwestern Live Cell Imaging Facility. Digital images (1024 × 1024 pixels; 8bit) were captured using either the 20x or 63x (oil) objectives. Different combinations of false colors were assigned to each experiment for a better visualization. Fiji ImageJ (NIH) was used to generate projection of the Z-stacks to form a single tiff image. Adobe Photoshop CS5 was used to adjust digital image size, resolution, and contrast. All modifications were uniformly applied across images. Cells positive for *Agrp*, *Dbh*, TH, *Mc3r*, and Tomato were manually counted on digital images. Numbers of double labeled cells were included in histology figures. In addition, we evaluated brain sections stained for *Pomc* and *Agrp* transcripts. Because *Agrp* and *Pomc* signals within Arc neurons were often too strong to distinguish individual RNAScope dots, instead, we estimated the total amount of signals per profile in Image J. Each cell body was outlined and the Mean measurement was applied. In peripheral tissues, *Agrp* expression was evaluated in combination with TH immunoreactivity. TH stains allowed us to delineate individual sympathetic, glomus type I, and adrenal cells. Each cell was outlined and the number of *Agrp* dots per profile were counted on digital images. Data from normoxia and hypoxia groups were expressed as mean values (±s.e.m) and analyzed using a t-test with P < 0.05.

## Appendix A Supplementary data

Supplementary figure 1 (**A**) Mapping of AgRP immunoreactivity (FastRed) in tissues of wild-type mice. AgRP-positive elements were seen in the paraventricular hypothalamus and adrenal medulla. Only faint immunoreactivity was seen in the SCG and none in the NG. (**B**) Chromogenic RNAScope for *Agrp* (red dots) in tissues of wild-type mice. SCG and NG contained Agrp-expressing neurons, but not the CMG and colon. All tissues were counterstained with hematoxylin. (**C**) *Agrp*-driven Cre activity in the Arc and colonic wall of the Agrp-Cre-tdTomato mouse. Tomato was never seen in any other sites including the CB and SCG. (**D**) Table summarizing the distribution patterns of Agrp-driven Cre activity, *Agrp* mRNA (RNAScope) and AgRP immunoreactivity across mouse tissues. A total of different 3 mice for each staining. Arc and CB were the only tissues with both mRNA and peptide. Surprisingly, the SCG and NG contained *Agrp* transcript, but little immunoreactivity. Conversely, the adrenal medulla contained abundant immunoreactivity but only little *Agrp* mRNA. Additional images of this anatomical survey can be found in Supplementary Fig. 1. Qualitative estimates were made by considering both signal strength and the number of labeled cells: ++++, highest; +++, high; ++, moderate; +, low; +/−, inconsistent or weak signals; –, absent. Abbreviations: 3V, third ventricle; Adx, adrenal gland (medulla); Arc, arcuate nucleus; CMG, celiac mesenteric ganglion; SCG, superior cervical ganglion; me, median eminence; NG, nodose ganglion. **Supplementary figure 2.** Collection of additional immunostainings performed on PPFE tissue sections from human cadavers. AgRP staining convincingly labeled cells in the adrenal gland and the carotid bodies of 3 female individuals. **Supplementary figure. 3.** Plasma levels of insulin, leptin, TNFα, glucose, and lactate from two groups of mice submitted to either normoxia or hypoxia (10%) for 48 h. Data are mean values (s.e.m) and analyzed with unpaired T-test. Glucose and lactate were measured using contour blood glucose monitoring system (Bayer, model 7151G) and lactate plus system (Nova, model 62624). At the end of the experiment blood was collected in EDTA tubes (Sarstedt Inc 16.444.100). Plasma was isolated by centrifugation (4000 g × 15 min at 4 °C) and was stored at −80 °C until analysis. Plasma leptin (Mouse Leptin ELISA Kit, Crystal Chem, 90030), insulin (Mouse Ultrasensitive Insulin ELISA Kit, Crystal Chem, 90080), and TNF-alpha (TNF alpha Mouse ELISA Kit, Invitrogen, BMS607-3) were measured as specified by the vendors. **Supplementary figure 4.** (**A, B**) Agrp expression in the Arc of a mouse fed ad libitum or fasted for 24 h. RNAScope (red) revealed robust elevation of Agrp expression in the food deprived mouse. (**C–F**) In the same animals, RNAScope (red) revealed no discernable changes in *Agrp* levels of the CB. Estimates of Agrp signals (3 mice/group) revealed no significant changes in expression levels in CB. Tissues were counterstained with TH (green) to delineate glomus cells. (**G, H**) RNAscope for *Agrp* (red) and *Lepr* (green) in the CB of a wild-type mouse. Note that Lepr-expressing cells (white arrows) are clearly visible in structures resembling blood vessels surrounding glomus cells, but not in glomus cells themselves. The tissue was counterstained with DAPI (grey). Scale bar in A applies to B, C, D, and G. Scale bar in E applies to F and H. Abbreviations: Arc, arcuate nucleus; CA, carotid artery; CB, carotid body; SCG, superior cervical ganglion. Supplementary figure 1
