# Supplementary material for: Hypoxia inducible factor-dependent upregulation of Agrp in glomus type I cells of the carotid body
Source: Mol Metab. 2025 Jan 8;92:102095. doi: 10.1016/j.molmet.2025.102095 (PMC11786784; doi:10.1016/j.molmet.2025.102095)

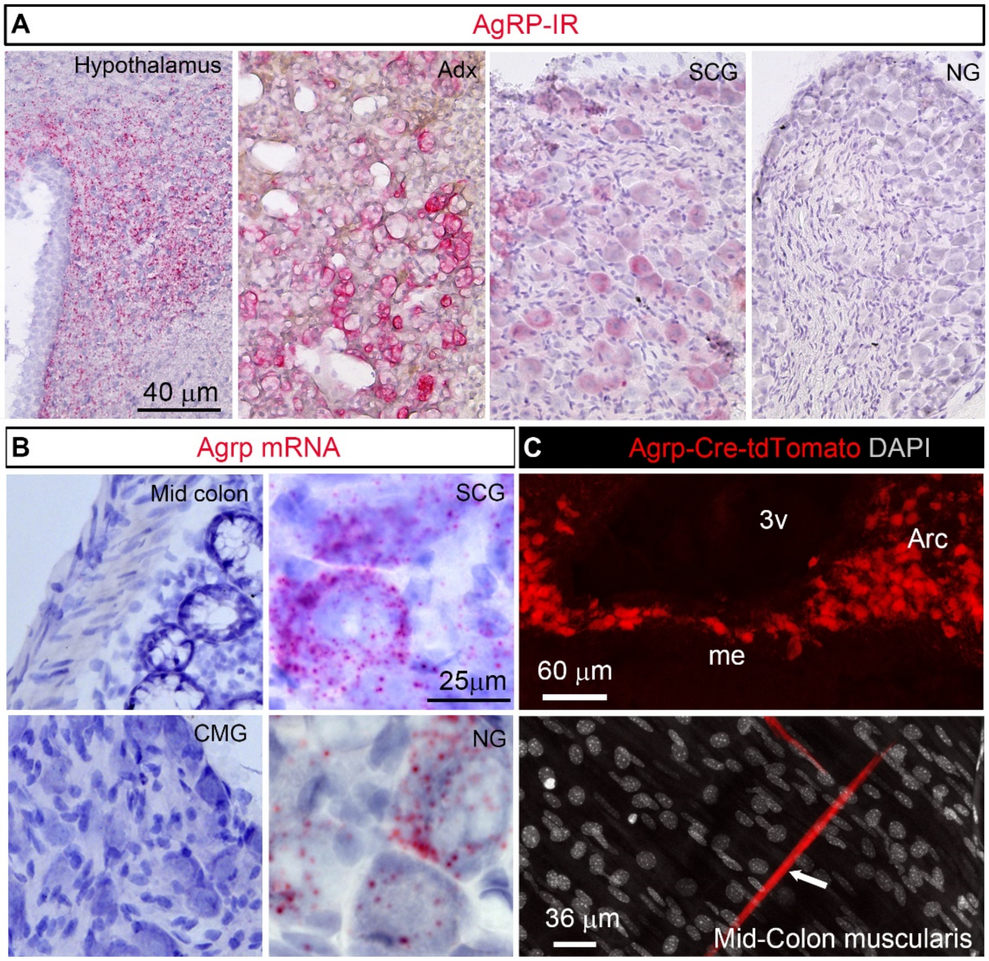

D

Structures	Agrp-Cre-tdTomato	Agrp mRNA	AgRP-IR	Comments
**Hypothalamus**	++++	++++	++++	
**Adrenal medulla**	-	+	+++	Identified as TH^+
**Carotid body**	-	+++	+++	Cells identified as TH^+/Dbh^- cells
**Superior cervical ganglion**	-	+++	+/-	Cells identified as TH^+/Dbh^+ neurons. Somas were faintly labeled by AgRP antibody
**Colon**	+/-	-	-	Sparse Tomato cells resembling smooth muscle
**Dorsal root ganglion**	-	-	-	
**Nodose ganglion**	-	+	-	~12% of vagal afferents positive for Agrp mRNA
**Celiac ganglion**	-	-	-	
**Lungs**	-	+/-	-	
**Testis**	+/-	+	-	

Supplementary Figure 1.

Supplementary Figure 2

Adrenal gland

Results AgRP staining:
Although adrenal sections showed a substantial general background staining, cells with a strong AgRP-immune reactivity (IR) could be easily distinguished. This AgRP-IR occurred in two types of patterns:
1) The medulla contains cells with a homogenous pink stained cytoplasm (green encircled cells).
2) Throughout the whole adrenal gland (both medulla and cortex) cells were present that showed a more granular staining pattern.

According to the literature AgRP expression is only expected in a selection of medullary cells. The images presented in the literature show cells with a homogenous stained cytoplasm comparable to the cells that were observed in the medulla. The granular staining AgRP staining pattern was not expected and might be explained by the fact that cells with a certain post-mortem interval start to express this protein due to the fact that they become hypoxic.

Adrenal gland sections without incubation with the primary antibody were used as negative controls. No staining was observed.

Postmortem interval of the used adrenal gland → 35 hr

(continued)

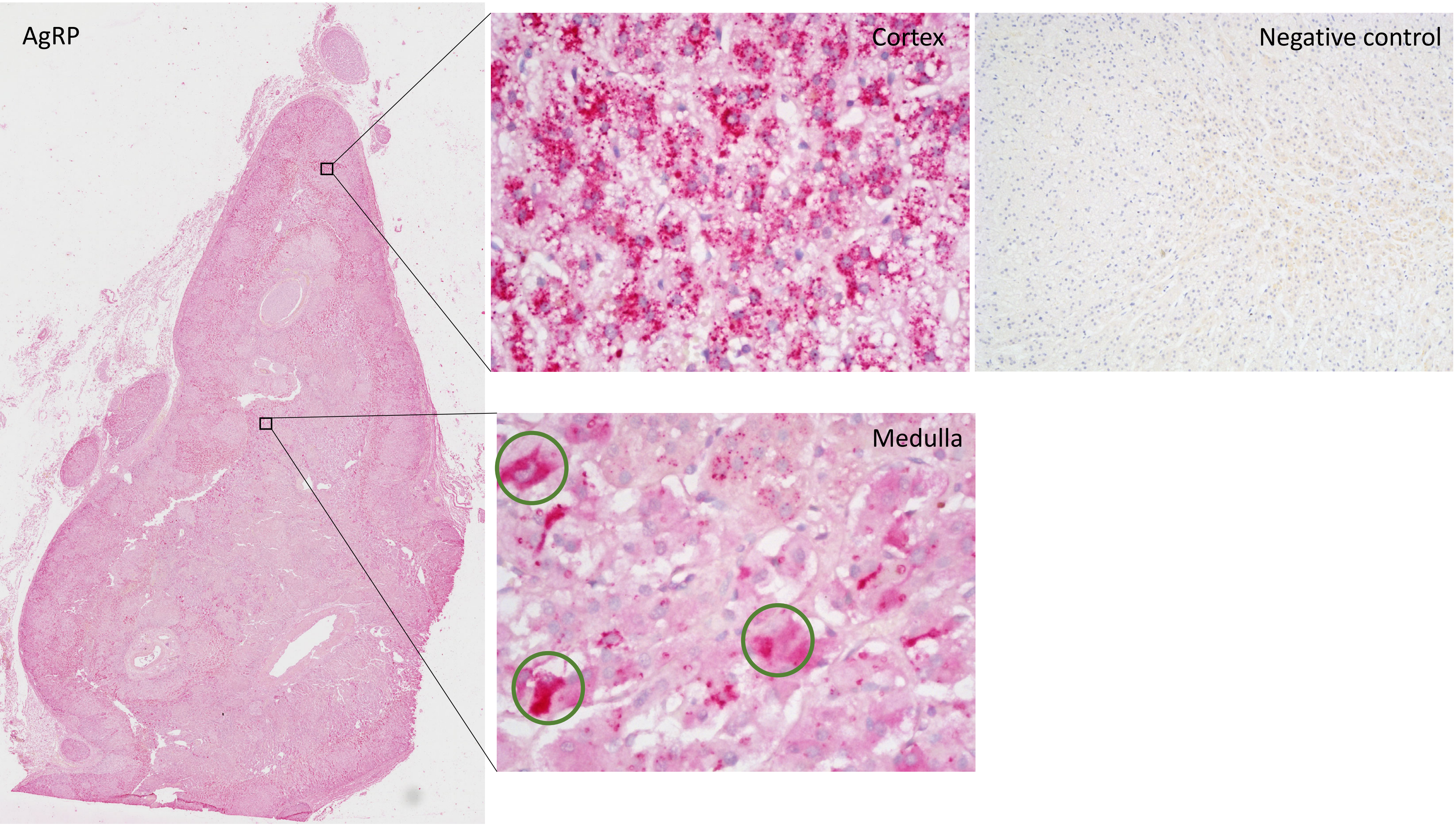

(continued)

Carotid body

General results:
All sections showed a substantial background staining (comparable to the adrenal gland).
Some very intense stained glomus cells were present. Occasionally these cells showed a homogenous staining, but most often the staining showed a granular pattern.

Cadaver #	Side of resected CB	Post Mortem Interval	Age	Sex	Formaldehyde fixation
1	Left	24 hours	82	F	Whole body perfusion
2	Right	44 hours	87	F	Whole body perfusion
3	Right	12 hours	87	F	Whole body perfusion

(continued)

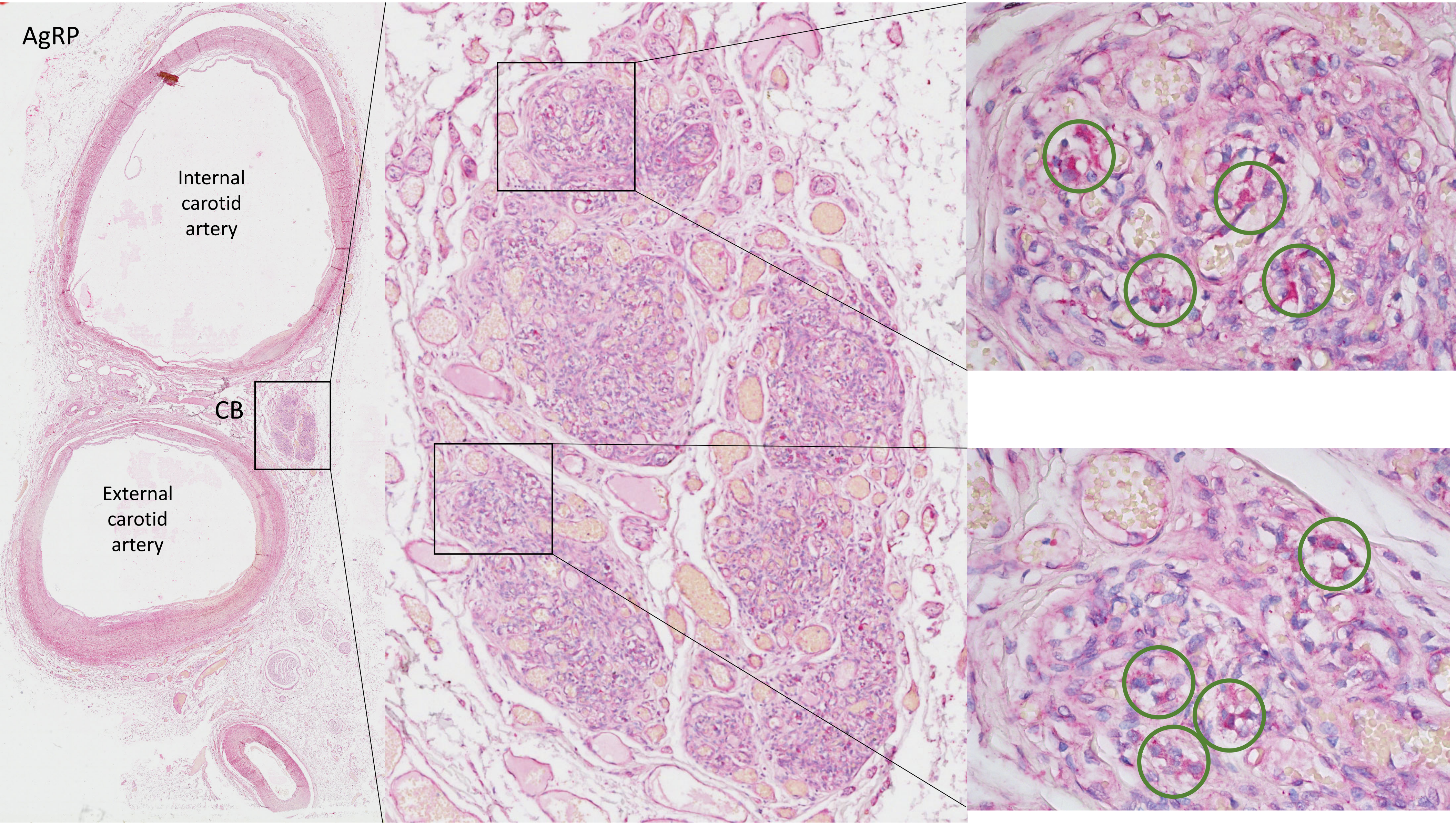

(continued)

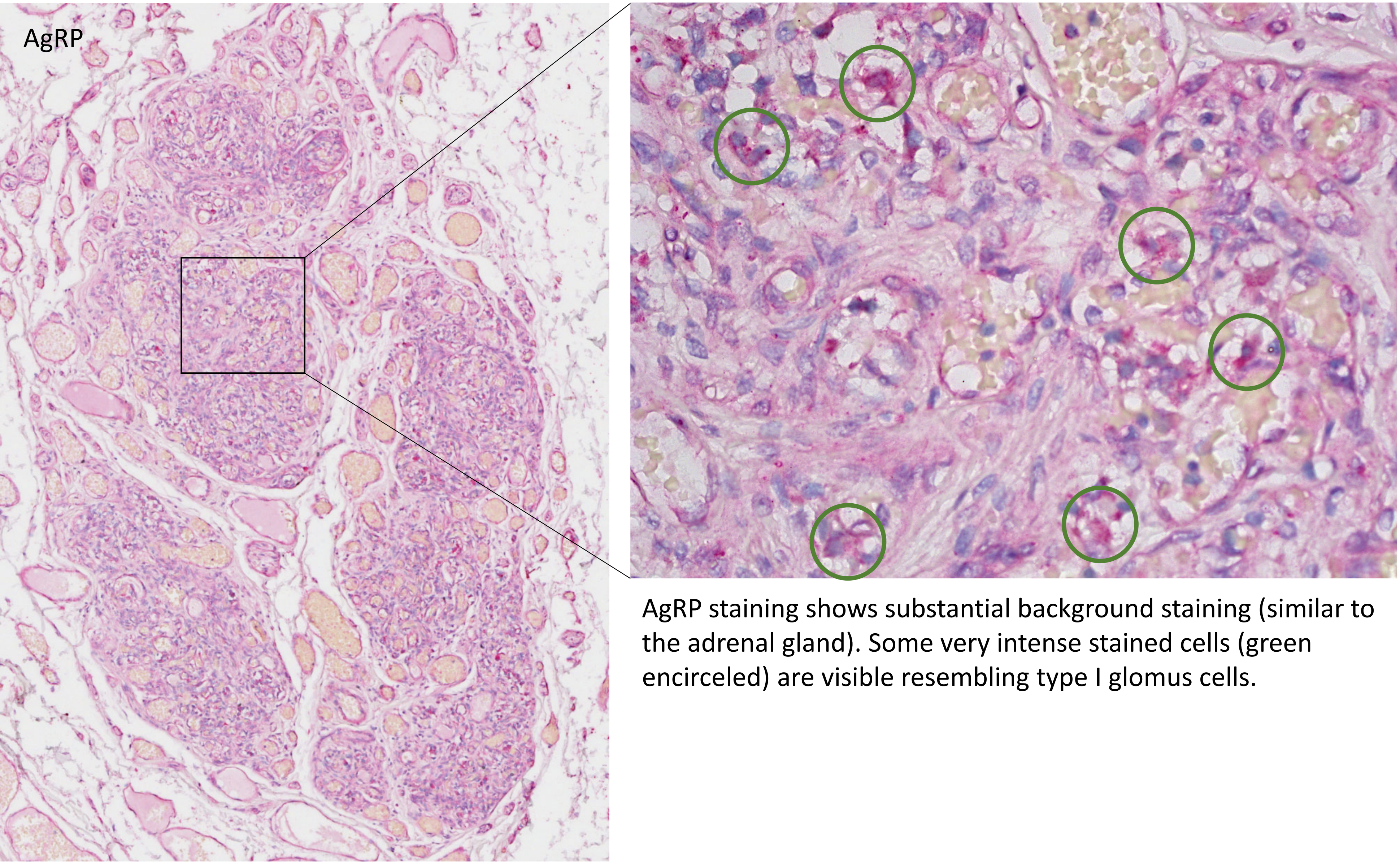

AgRP staining shows substantial background staining (similar to the adrenal gland). Some very intense stained cells (green encirceled) are visible resembling type I glomus cells.

(continued)

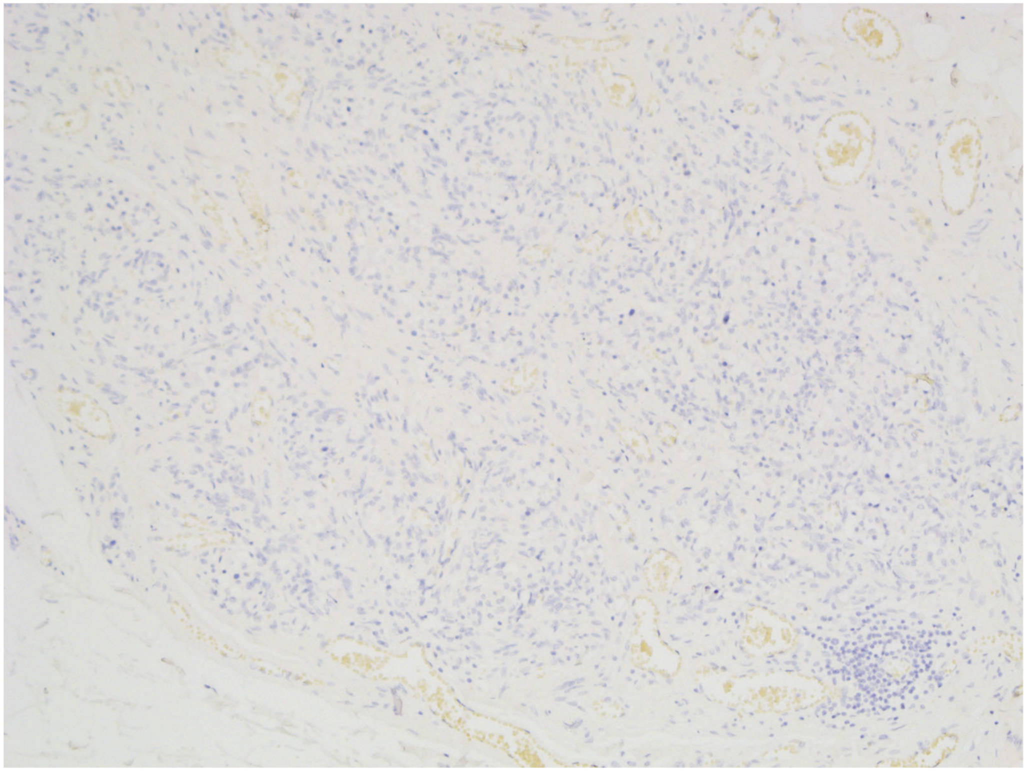

Negative control

(continued)

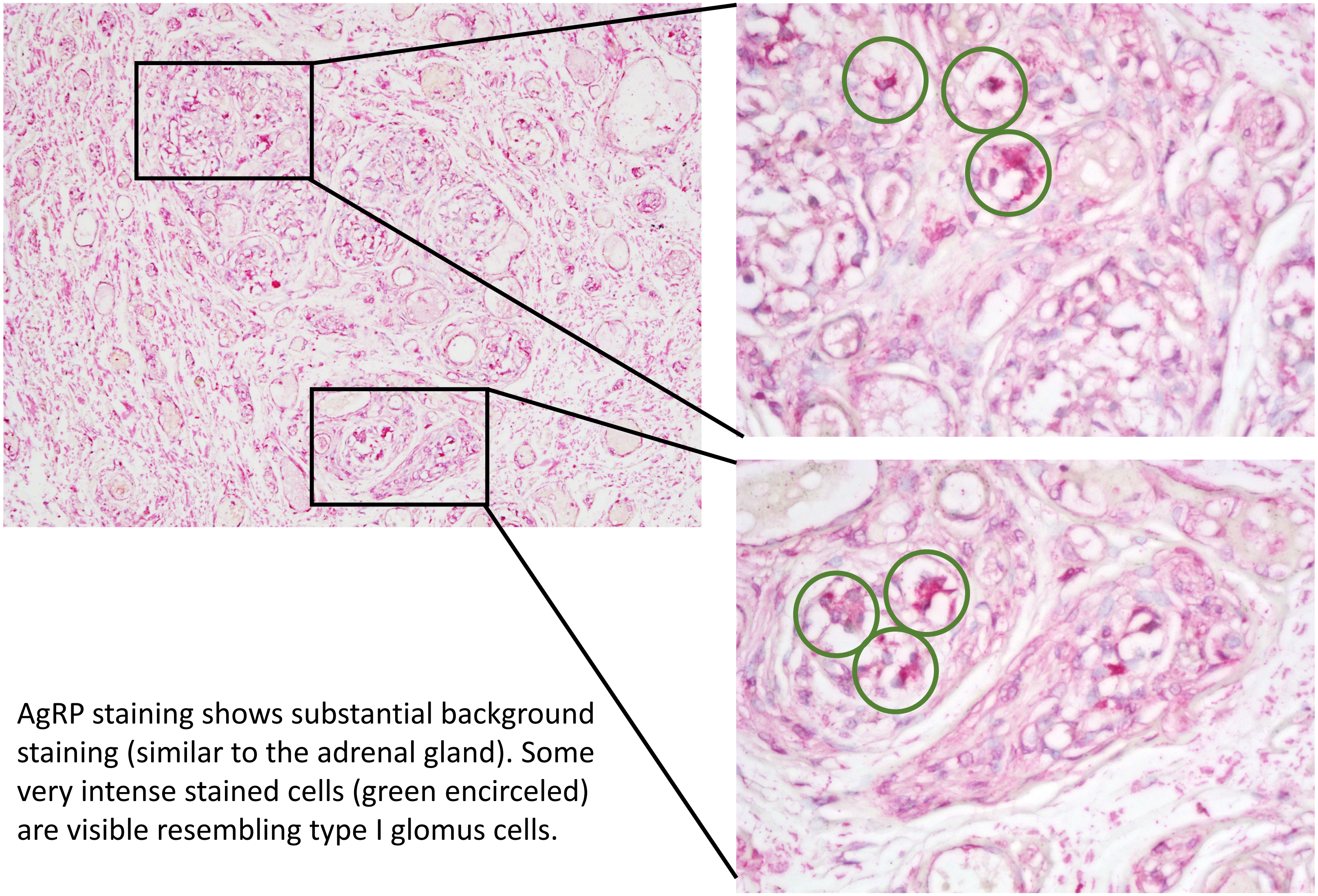

AgRP staining shows substantial background staining (similar to the adrenal gland). Some very intense stained cells (green encirceled) are visible resembling type I glomus cells.

(continued)

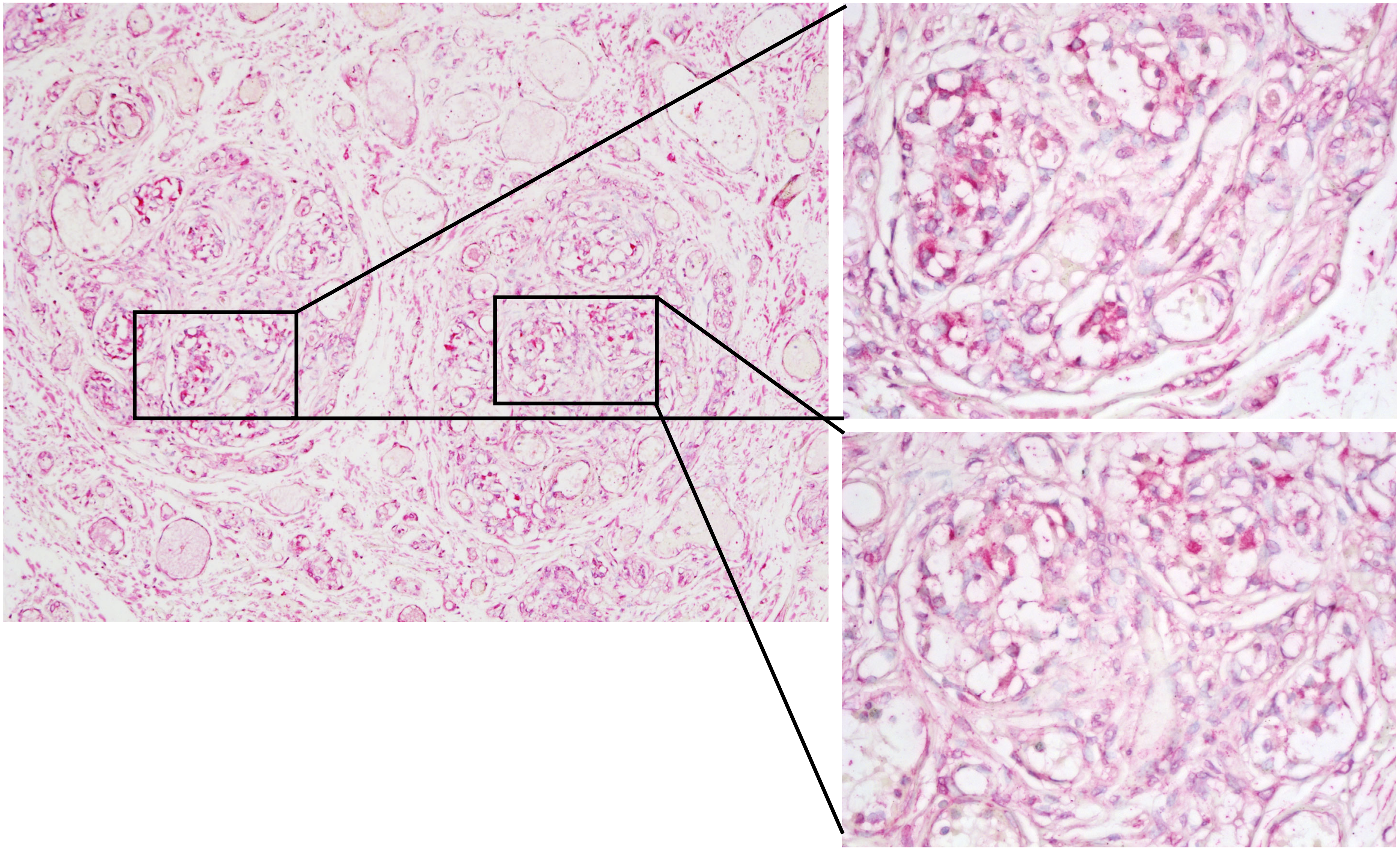

(continued)

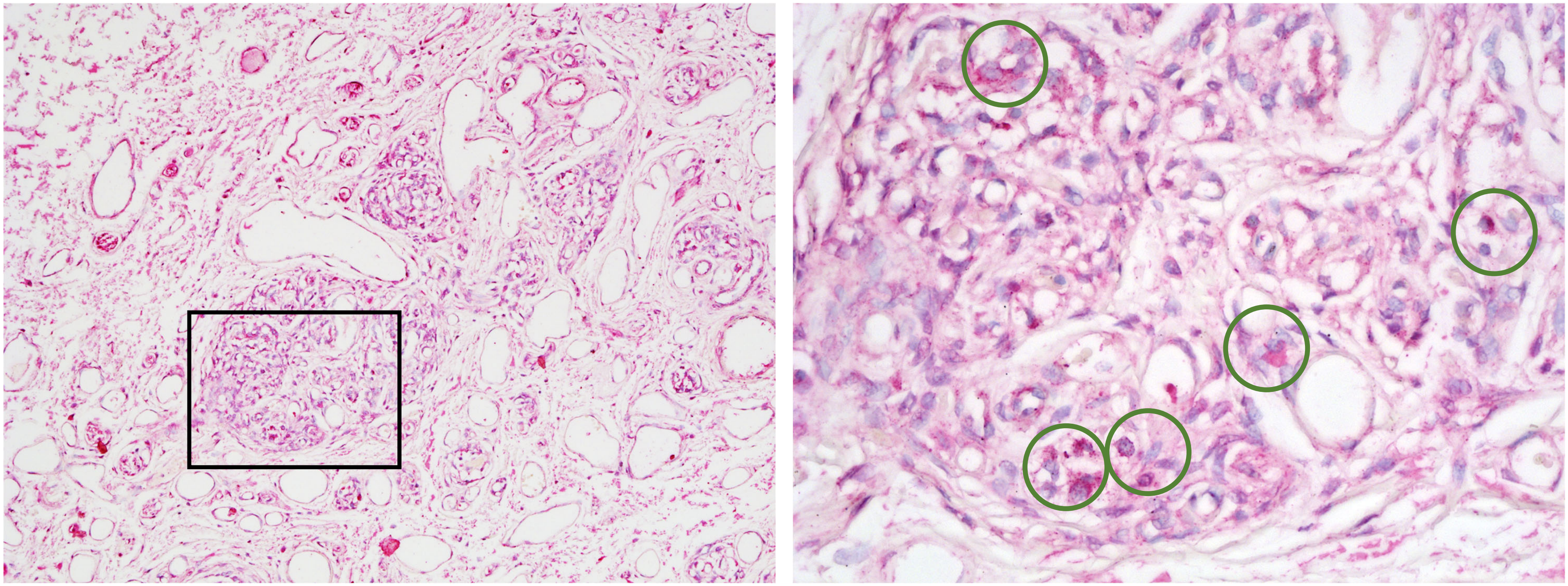

AgRP staining shows strong background staining (hinger compared to the adrenal gland and other CBs). Some very intense stained cells (green encirceled) are visible resembling type I glomus cells.

(continued)

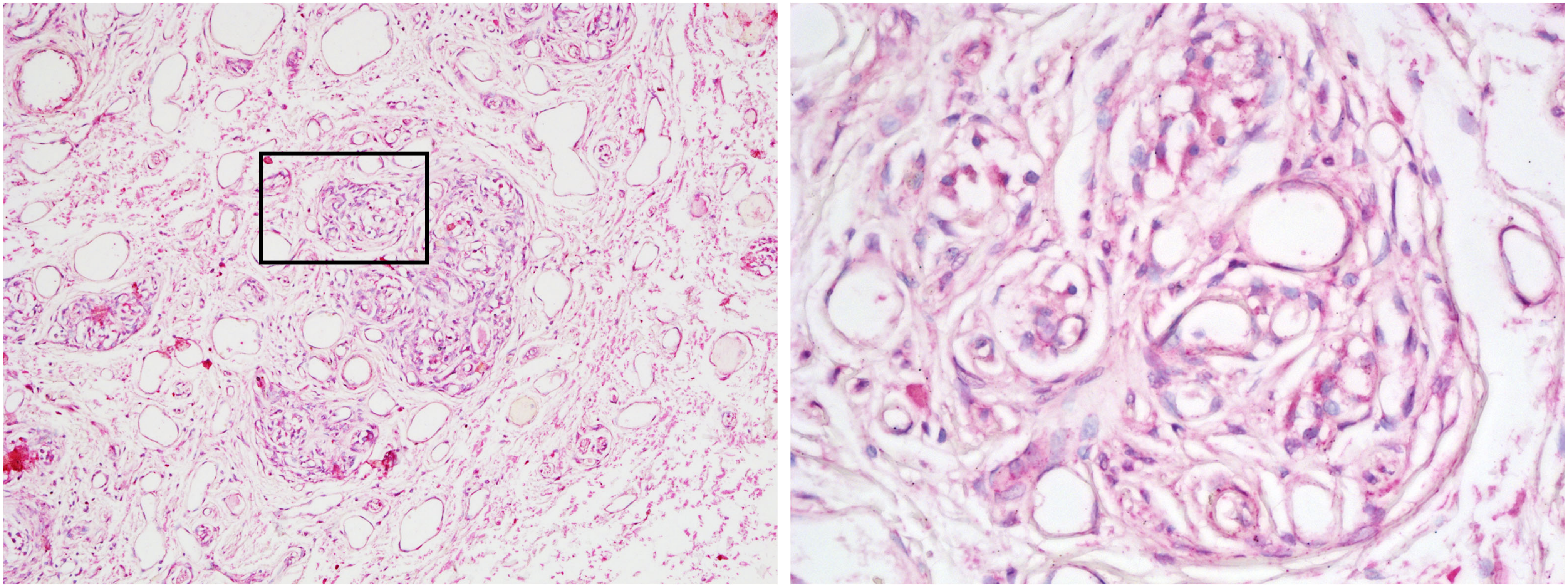

Supplementary figure 3

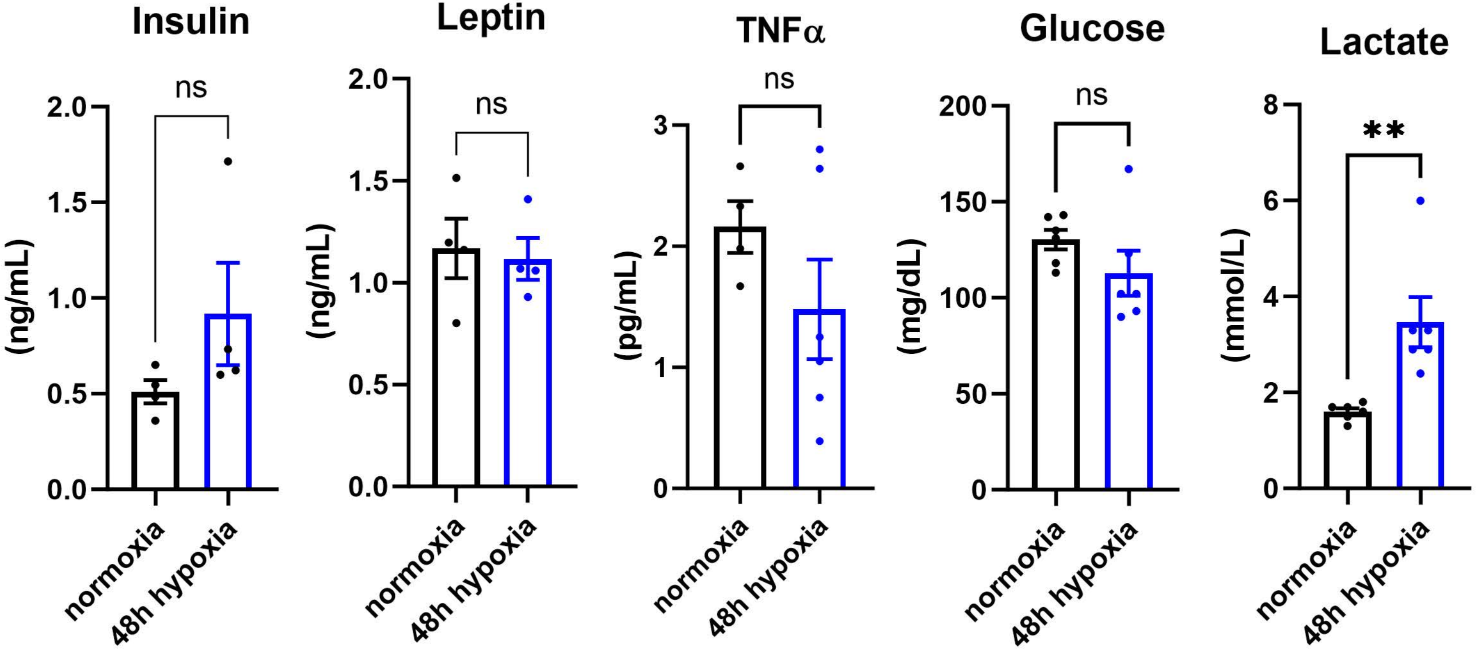

Supplementary Figure 4

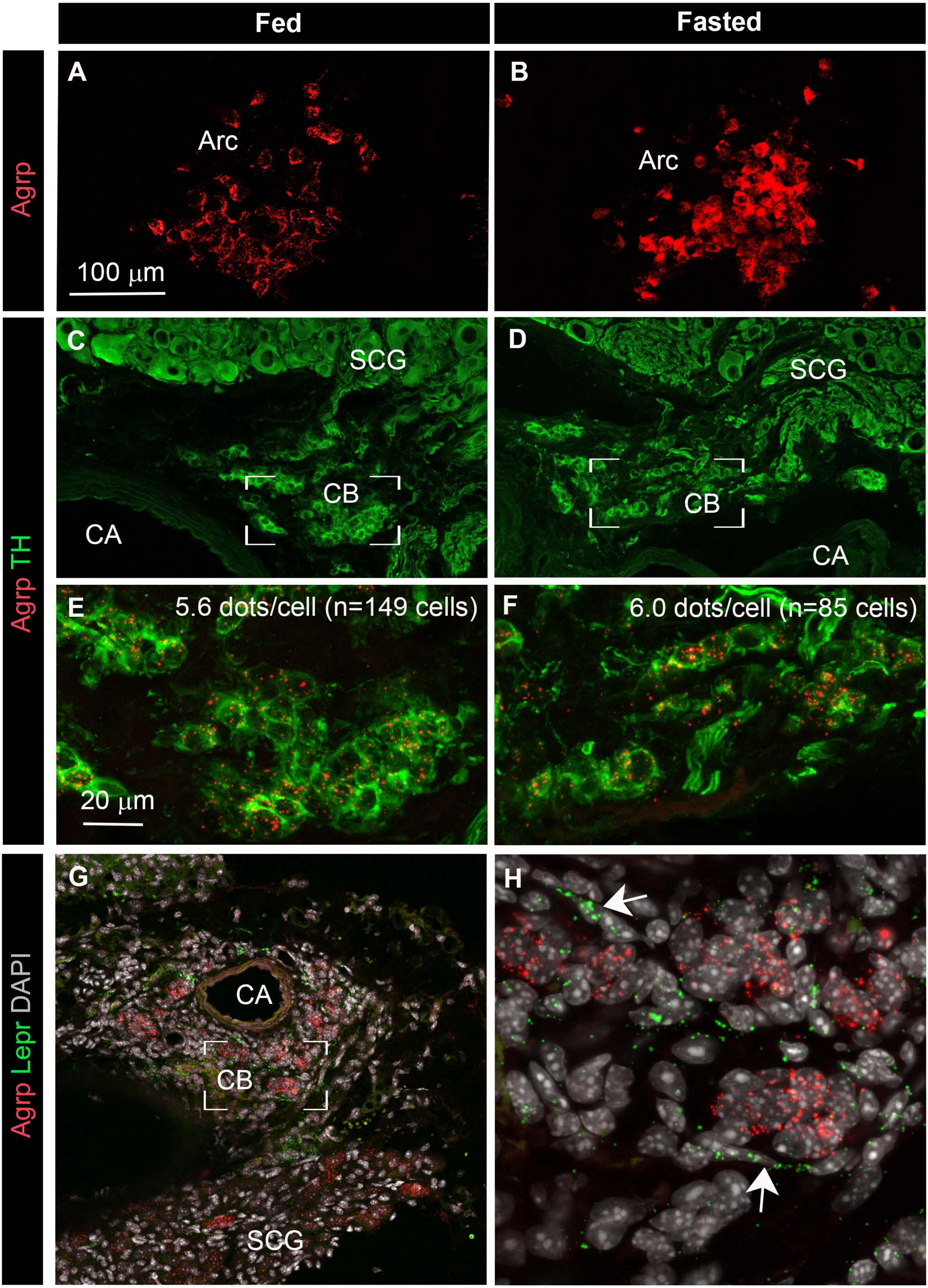

Supplement: Supplementary figure 1 — (A) Mapping of AgRP immunoreactivity (FastRed) in tissues of wild-type mice. AgRP-positive elements were seen in the paraventricular hypothalamus and adrenal medulla. Only faint immunoreactivity was seen in the SCG and none in the NG. (B) Chromogenic RNAScope for Agrp (red dots) in tissues of wild-type mice. SCG and NG contained Agrp-expressing neurons, but not the CMG and colon. All tissues were counterstained with hematoxylin. (C) Agrp-driven Cre activity in the Arc and colonic wall of the Agrp-Cre-tdTomato mouse. Tomato was never seen in any other sites including the CB and SCG. (D) Table summarizing the distribution patterns of Agrp-driven Cre activity, Agrp mRNA (RNAScope) and AgRP immunoreactivity across mouse tissues. A total of different 3 mice for each staining. Arc and CB were the only tissues with both mRNA and peptide. Surprisingly, the SCG and NG contained Agrp transcript, but little immunoreactivity. Conversely, the adrenal medulla contained abundant immunoreactivity but only little Agrp mRNA. Additional images of this anatomical survey can be found in Supplementary Fig. 1. Qualitative estimates were made by considering both signal strength and the number of labeled cells: ++++, highest; +++, high; ++, moderate; +, low; +/−, inconsistent or weak signals; –, absent. Abbreviations: 3V, third ventricle; Adx, adrenal gland (medulla); Arc, arcuate nucleus; CMG, celiac mesenteric ganglion; SCG, superior cervical ganglion; me, median eminence; NG, nodose ganglion. Supplementary figure 2. Collection of additional immunostainings performed on PPFE tissue sections from human cadavers. AgRP staining convincingly labeled cells in the adrenal gland and the carotid bodies of 3 female individuals. Supplementary figure. 3. Plasma levels of insulin, leptin, TNFα, glucose, and lactate from two groups of mice submitted to either normoxia or hypoxia (10%) for 48 h. Data are mean values (s.e.m) and analyzed with unpaired T-test. Glucose and lactate were measured using cont [file mmc1.pdf]
